# Biomarkers for Pulmonary Inflammation and Fibrosis and Lung Ventilation Function in Chinese Occupational Refractory Ceramic Fibers-Exposed Workers

**DOI:** 10.3390/ijerph15010042

**Published:** 2017-12-27

**Authors:** Xiaojun Zhu, Yishuo Gu, Wenjun Ma, Panjun Gao, Mengxuan Liu, Pei Xiao, Hongfei Wang, Juan Chen, Tao Li

**Affiliations:** 1 National Institute of Occupational Health and Poison Control, Chinese Center for Disease Control and Prevention, No. 29 Nanwei Road, Xicheng District, Beijing 100050, China; happyzhuxj@163.com (X.Z.); liumengxuan@niohp.chinacdc.cn (M.L.); woodyco@sina.com (P.X.); afei3669@163.com (H.W.); 2 Department of Occupational and Environmental Health Sciences, School of Public Health, Peking University, Beijing 100191, China; guyishuo@bjmu.edu.cn (Y.G.); mawenjun@bjmu.edu.cn (W.M.); gaopj@bjmu.edu.cn (P.G.); chenjuan94@bjmu.edu.cn (J.C.)

**Keywords:** refractory ceramic fibers (RCFs), pulmonary ventilation function, transforming growth factor-β1 (TGF-β1), ceruloplasmin (CP), forced vital capacity (FVC), forced expiratory volume in 1 s (FEV_1_)

## Abstract

Refractory ceramic fibers (RCFs) can cause adverse health effects on workers’ respiratory system, yet no proper biomarkers have been used to detect early pulmonary injury of RCFs-exposed workers. This study assessed the levels of two biomarkers that are related to respiratory injury in RCFs-exposed workers, and explored their relations with lung function. The exposure levels of total dust and respirable fibers were measured simultaneously in RCFs factories. The levels of TGF-β1 and ceruloplasmin (CP) increased with the RCFs exposure level (*p* < 0.05), and significantly increased in workers with high exposure level (1.21 ± 0.49 ng/mL, 115.25 ± 32.44 U/L) when compared with the control group (0.99 ± 0.29 ng/mL, 97.90 ± 35.01 U/L) (*p* < 0.05). The levels of FVC and FEV_1_ were significantly decreased in RCFs exposure group (*p* < 0.05). Negative relations were found between the concentrations of CP and FVC (B = −0.423, *p* = 0.025), or FEV_1_ (B = −0.494, *p* = 0.014). The concentration of TGF-β1 (B = 0.103, *p* = 0.001) and CP (B = 8.027, *p* = 0.007) were associated with respirable fiber exposure level. Occupational exposure to RCFs can impair lung ventilation function and may have the potential to cause pulmonary inflammation and fibrosis. TGF-β1 and CP might be used as sensitive and noninvasive biomarkers to detect lung injury in occupational RCFs-exposed workers. Respirable fiber concentration can better reflect occupational RCFs exposure and related respiratory injuries.

## 1. Introduction

Refractory ceramic fibers (RCFs), also termed as aluminosilicate wools (ASW), are amorphous fibers that belong to a class of materials, termed man-made vitreous fibers (MMVFs), which also include glass wool, rock (stone) wool, slag wool, and special-purpose glass fibers [1,2]. RCFs have certain desirable properties, including high tensile strength, flexibility, low thermal conductivity, light weight, and low heat storage, which enable this material to be widely applied in industries, such as steel, foundry, and forging [3,4].

However, adverse health effects of occupational exposure to RCFs have raised concern in recent years for the fibers’ respirable size and relatively high biopersistence [5]. Respirable fibers of RCFs, particularly those exceeding the diameter of pulmonary macrophage (14 to 21 μm), could result in macrophage impairment and other lung irritation [6]. Epidemiological studies revealed that occupational exposure to RCFs may increase the incidence rate of respiratory symptoms, respiratory dysfunction, and pleural plaques [1,7]. In 1988 and 2002, the International Agency for Research on Cancer (IARC) classified RCFs as a 2B carcinogen (possibly carcinogenic to humans) twice based on sufficient animal studies and limited epidemiological data [8]. 

There are two different methods of describing the concentration of RCFs: total dust concentration (mg/m^3^) and respirable fiber concentration (f/cm^3^) [9]. Respirable fibers are defined as particles >5 μm long, <3 μm width and with a length to diameter ratio of >3:1 [10]. Total dust includes respirable fibers and other particles. The two kinds of dust may cause different health effects. Therefore, these two kinds of concentration were measured simultaneously in this study, and were combined to evaluate the exposure features of RCFs-exposed workers.

Chest X-ray examination and pulmonary function test are commonly used for checking up on occupational respiratory injuries [11]. The indicators of lung function assessment, such as forced vital capacity (FVC), forced expiratory volume in 1 s (FEV_1_), and FEV_1_:FVC ratio (FEV_1_/FVC) are still used to evaluate the possible lung injury caused by RCFs exposure in health checkup. However, neither chest X-ray examination nor pulmonary function test is sensitive enough to perceive early health effects. These examinations could be modified by many confounding factors, especially noncompliance of workers and unprofessional determination [12]. Therefore, it is necessary to investigate proper biomarkers for lung injury among RCFs-exposed workers. Previous epidemiological studies usually focused on the carcinogenic effect of RCFs, while studies concerning pulmonary inflammation and fibrosis of the RCFs-exposed workers are still insufficient. There are some sensitive biomarkers, including transforming growth factor-β1 (TGF-β1) and ceruloplasmin (CP), which had been found able to detect the pulmonary inflammation and fibrosis induced by occupational exposure to asbestos or other toxicants. TGF-β1 is a multifunctional cytokine that is critically involved in the pathogenesis of fibrosis and contributes to the influx and activation of inflammatory cells [13,14]. Previous studies found that TGF-β1 plays a key role in the development of lung fibrosis, and the expression of TGF-β1 increases in lung tissue of patients with lung fibrosis and animal models of pulmonary fibrosis [15,16,17]. Asbestos may induce the expression of TGF-β1 in the interstitial lung disease murine modal [18]. The rise of serum CP level was observed in workers with silicosis or anthrasilicosis, and is associated with the progression of anthrasilicosis [19,20]. The rise of serum CP level might be used as a signal of the progression of pulmonary fibrosis and a complimentary factor that is associated with inflammatory conditions [21]. It is urgent to find out workers’ pulmonary injuries at early stage through non-invasive detection by the measurement of biological markers rather than clinical assessment.

The objective of this study was to evaluate whether TGF-β1 and CP could be used as biomarkers to detect lung injury and investigate their relationships with the indicators of lung ventilation function among RCFs-exposed workers. 

## 2. Materials and Methods

### 2.1. Study Design and Subjects

This cross-sectional study was performed in a RCFs factory in Shandong province for three reasons: (1) the factory has two branch plants located separately: one is RCFs-related while the other is RCFs-free; (2) it offers annual health checkup for employees, which enables us to collect specimens and minimize the interference with normal work schedules; and (3) we have cooperated with this factory for years and the workers are compliant. 

A total of 374 participants were investigated in this study. The inclusion criteria for all the subjects included: (1) at least had one year of employment in the factory and stayed six months in the same work location; and (2) aged from 20 to 50 years. Then, 31 workers were excluded because they failed the lung function test or could not provide serum samples, or their questionnaires were lack of essential information. Finally, 343 subjects were involved in this survey. 172 subjects from six different RCFs-related workshops (including four manufacturing workshops, one processing workshop, and one module workshop) were defined as the RCFs-exposed group, while 171 subjects without exposure to RCFs or other occupational toxicants in the same factory were defined as the control group. Some workers in the RCF-exposed groups wore gauze masks and cotton gloves as protective equipment. 

The ethical approval of this study was approved by the Medical Ethics Committee of National Institute of Occupational Health and Poison Control, Chinese Center for Disease Control and Prevention, Beijing, China (record number: 201407, 29 April 2014).

### 2.2. Exposure Assessment

Occupational exposure assessments of RCFs were performed in June 2015. There were six RCFs-related workshops in this factory: No. 1 to No. 4 manufacturing workshops, processing workshop, and module workshop. A total of 23 jobs were identified throughout the six workshops. For jobs that have fixed operational area, area sampling method was used for exposure measurement; for jobs that have unfixed operational area, personal sampling method was used for exposure measurement. Total dust concentration (mg/m^3^) and respirable fiber concentration (f/cm^3^) were measured simultaneously for all RCFs-related job categories in these six workshops. Samples of total dust were collected and measured according to the national criteria: GBZ/T192.1-2007 (Determination of dust in the air of workplace-Part 1: Total dust concentration). The method in these criteria is using a sampling pump to pull air though a filter that traps suspended particles. The mass of total dust on the filter is measured using gravimetric analysis, and total dust concentration is determined as the ratio of the total dust mass to the volume of air sampled, reported as mg/m^3^. Samples of respirable fiber were collected and analyzed according to the World Health Organization reference method with phase contrast optical microscopy [10]. Total dust sampling was performed by Leland Legacy sample pump (SKC, Covington, GA, USA) with 37 mm perchloroethylene filter membrane (Beijing Municipal Institute of Labour Protection, Beijing, China) and in 10 L/min sampling flow rate. Respirable fiber sampling was performed by Apex2 Air sampling pump (Casella, Bedford, UK) with 25 mm mixed cellulose ester (MCE) filters (SKC, Inc., Eighty Four, PA, USA) and in 2 L/min sampling flow rate. All of the collection media were positioned within the workers’ breathing zone. Air sampling of each job was performed continuously for at least three full shifts during normal working hours. 

Based on the occupational health survey, 40-h (a workweek) time-weighted average concentration (C*_TWA_*_, 40 h_) in different jobs was calculated by the following formula: C*_TWA_*_, 40 h_ = C_s_ × (t_w_/40) (C_s_: measured concentration of total dust or respirable fiber in each work category; t_w_: actual work time per week of workers in the same work category). The C*_TWA_*_, 40 h_ of each job was used to represent the exposure level of a group of workers who were at that time occupied in the same job. Each worker’s exposure was derived from the C*_TWA_*_, 40 h_ of the job that they belonged to at that time.

According to the existing standards and recommendations, C*_TWA_*_, 40 h_ of total dust <5.0 mg/m^3^ or ≥5.0 mg/m^3^ are defined as low or high total dust exposure level, respectively, and C*_TWA_*_, 40 h_ of respirable fiber <0.5 f/cm^3^ or ≥0.5 f/cm^3^ are defined as low or high fiber exposure level, respectively. These two kinds of concentration were taken into account together to evaluate workers’ exposure features. According to the measurement results, RCFs-exposed workers were divided into three exposure subgroups: exposure to low fiber level and low total dust level (LL), exposure to low fiber level and high total dust level (LH), and exposure to high fiber level and high total dust level group (HH). 

### 2.3. Questionnaires and Lung Function Test

All of the subjects voluntarily joined this study with informed consents, and then answered a questionnaire conducted by well-trained investigators face to face. The questionnaire included their occupational history, personal history, smoking status, and other demographic information.

Lung function tests were performed at the beginning of the work shift among subjects by using Carefusion™ MasterScreen Pneumo (CareFusion, San Diego, CA, USA, ERS/ATS), according to its instruction manual, after the age and height of subjects were measured. Forced vital capacity (FVC), forced expiratory volume in 1 s (FEV_1_), and FEV_1_:FVC ratio (FEV_1_/FVC) were chosen as lung function indicators in this study. All of the indicators were showed and analyzed as the percentage of the measured value on the predicted value. Individual predicted values of these indicators were calculated with equations that were developed by the European Respiratory Society (ERS) in 1993 (equations were showed in Appendix A, Table A1).

### 2.4. Specimens Collection and Determination of Biomarkers’ Level

Blood samples were collected after workers finished the lung function tests. Peripheral blood (3 mL) were drawn from each subject, then collected in a covered test tube with thromboplastic medicaments and allowed to clot by leaving it undisturbed at 5 °C for 1 h. Clot was then removed by centrifugation at 3000 rpm for 10 min in a refrigerated centrifuge. After centrifugation, the serum was immediately transferred into a clean polypropylene tube and preserved at −80 °C until analysis. 

The concentrations of TGF-β1 in serum were determined by commercially available ELISA kits (Boster, Wuhan, China) according to the instruction manual, and the optical density was measured by Thermo Scientific Varioskan Flash (Thermo Fisher Scientific, Waltham, MA, USA). The concentrations of CP in serum were determined by commercially kits (Jiancheng, Nanjing, China) and was measured by ultraviolet spectrophotometry (VIS-7220, Ruili Analytical Instrument Co., Ltd., Beijing, China) at 540 nm. Laboratory researchers were blind to the exposure status of the subjects.

### 2.5. Statistical Analysis

The data of questionnaires, lung function tests, and experiments were collected by using Epidata 3.1 (The EpiData Association, Odense, Denmark). All of the analyses were performed with SPSS19.0 (IBM, Armonk, NY, USA). The differences in continuous and categorical parameters among different RCFs-exposed subgroups and control group were, respectively, tested using the One-Way ANOVA and the χ^2^ test. The Pearson correlations and linear regressions were performed between biomarkers (TGF-β1, CP) and lung function indicators (FVC, FEV_1_, and FVC/FEV_1_). Multiple linear regression analysis (stepwise) was used to analyze the influence factors of biomarkers. Statistical differences of at least *p* < 0.05 were considered as statistically significant.

## 3. Results

### 3.1. RCFs Exposure Assessment

Table 1 shows the measurement results of C*_TWA_*_, 40 h_ of respirable fiber and total dust in the six RCFs-related workshops. Arithmetic mean and range of C*_TWA_*_, 40 h_ among all the job categories of different workshops were summarized in Table 1. According to the grouping rule mentioned in Section 2.2, 101 RCFs-exposed workers were exposed to low fiber level and low total dust level (LL); 39 RCFs-exposed workers were exposed to low fiber level and high total dust level (LH); 32 RCFs-exposed workers were exposed to high fiber level and high total dust level (HH).

### 3.2. General Information

172 RCFs-exposed workers and 171 controls were finally involved in this study. RCFs-exposed workers were divided into three subgroups (LL, LH, and HH) according to their different exposure features. The main characteristics of the participants were summarized in Table 2. Significant differences of age and gender were observed among these four groups (*p* < 0.001). The average age of HH group (37.88 ± 5.49) was significantly higher than other three groups (*p* < 0.001). There was no significant difference in smoking status among these four groups (*p* = 0.077). According to the questionnaires and clinical examination, workers that engaged in this survey did not develop fever or other clinical diseases.

### 3.3. Determination of Indicators in Serum and Lung Function

As shown in Table 2, the levels of TGF-β1 and CP were significantly higher, whereas the levels of FVC and FEV_1_ were significantly lower in the RCFs-exposed groups than those in the control group. The level of TGF-β1 and CP increased with the extent of RCFs exposure (*p* < 0.05), and significantly increased in the HH group (1.21 ± 0.49 ng/mL, 115.25 ± 32.44 U/L) compared with the control group (0.99 ± 0.29 ng/mL, 97.90 ± 35.01 U/L) (*p* < 0.05). The levels of FVC and FEV_1_ decreased with the extent of RCFs exposure (*p* < 0.05) and significantly decreased in three RCFs exposure subgroups when compared with the control group (*p* < 0.05). FEV_1_/FVC also showed a slight decrease in RCFs exposure groups, but the differences were not significant.

### 3.4. Correlations between Biomarkers and Lung Function Indicators

**Figure 1:**
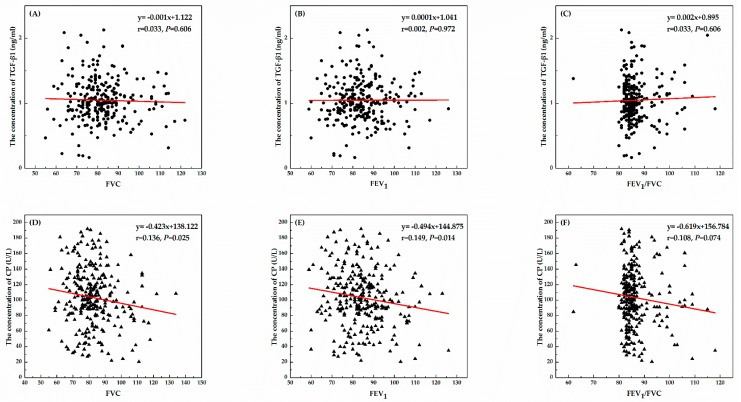
The correlations between TGF-β1 or CP and indicators of lung function (FVC, FEV_1_, and FEV_1_/FVC) among all subjects. (**A**) The correlation between TGF-β1 and FVC; (**B**) The correlation between TGF-β1 and FEV_1_; (**C**) The correlation between TGF-β1 and FEV_1_/FVC; (**D**) The correlation between CP and FVC; (**E**) The correlation between CP and FEV_1_; (**F**) The correlation between CP and FEV_1_/FVC.

The correlations between biomarkers and lung function indicators were assessed by regression analysis among all the subjects, and the results were shown in Figure 1. There were negative relationships between the concentrations of CP and FVC (B = −0.423, *p* = 0.025) or FEV_1_ (B = −0.494, *p* = 0.014), whereas the level of TGF-β1 was not significantly related to the indicators of lung function. 

### 3.5. Influential Factors of Biomarkers

Multiple linear regressions analyses were performed to detect the influential factors of the concentrations of TGF-β1 and CP in serum. In order to evaluate the respective effects of respirable fiber and total dust, fiber exposure level and total dust exposure level were separately included in the equations. Independent variables include fiber exposure level, total dust exposure level, age, gender, and smoking status. Variable assignments were shown in Appendix A (Table A2). Smoking habits and gender were, respectively, set as stratification factors to detect their possible effect on the association between RCFs exposure and biomarkers. Independent variables initially included in the stratified regressions modules were the above-mentioned five variables, except for the corresponding stratification factor. The results of multiple linear regressions were shown in Table 3. 

#### 3.5.1. TGF-β1

Among all of the subjects, the concentration of TGF-β1 was only associated with respirable fiber exposure level (B = 0.103, *p* = 0.001). In ever-smoking workers, respirable fiber exposure level (B = 0.245, *p* < 0.001) was found in association with TGF-β1 level. Both in male and female workers, only respirable fiber exposure level (B = 0.098, *p* = 0.010; B = 0.106, *p* = 0.043) was included in the final regression model.

#### 3.5.2. CP

Among all subjects, the level of CP was affected by respirable fiber exposure level (B = 8.027, *p* = 0.007), gender (B = −14.710, *p* = 0.001), and smoking status (B = 13.926, *p* = 0.002). Other factors were not included in the final regression model. In ever-smoking workers, a significant association between CP and RCFs exposure was not observed. However, respirable fiber exposure level (B = 7.238, *p* = 0.048) and gender (B = −15.751, *p* = 0.001) were found in association with CP in never-smokers. In male workers, the level of CP was associated with smoking habit (B = 14.334, *p* = 0.004); while in female workers, the level of CP was associated with respirable fiber exposure level (B = 10.006, *p* = 0.048).

## 4. Discussion

Since China has not yet set occupational RCFs exposure limits, existing standards and recommendations from foreign countries or international agencies were applied in this study to evaluate the exposure level. The National Institute for Occupational Safety and Health (NIOSH) recommended a exposure limit of 0.5 f/cm^3^ (C*_TWA_*_, 40 h_) in 2006 [22]. The Occupational Safety and Health Administration (OSHA) issued permissible exposure limit (PEL) of 0.5 f/cm^3^ for RCFs and 15 mg/m^3^ for total dust [23]. In the United Kingdom, the Health and Safety Commission of the Health and Safety Executive has implemented a maximum exposure limit of 1 f/cm^3^ for RCFs and of 5 mg/m^3^ for total dust [24]. In this study, the concentrations of total dust in processing workshop and module workshop exceeded the U.K. standard, but were still lower than the PEL of OSHA. The concentration of respirable fiber in module workshop exceeded the limits of both NIOSH and OSHA. Overall, the exposure level of workers in the module workshop was relatively higher. Effective measures should be taken to reduce the RCFs concentration in the workplaces. In present study, 0.5 f/cm^3^ and 5 mg/m^3^ was set as threshold values. Europe and America have reduced the REL and PEL of RCFs in recent years. However, due to the backward production technology and ineffective protection measures, the occupational exposure level of RCFs-related workers in China was clearly higher than America and other developed countries. Therefore, choosing 0.5 f/cm^3^ and 5 mg/m^3^ as threshold values can better reflect the actual exposure of Chinese RCFs-exposed workers. In this study, lung function tests were performed at the beginning of the work shift, and blood samples were collected after workers had finished the lung function tests. These examinations were performed during the normal workdays, and all of the workers stayed in the same work location at least for six months. Therefore, the results of lung function tests and the period of biological sampling may reflect the actual physical state of workers who have been continuously exposed to RCFs. Both FVC and FEV_1_ showed significant decline among RCFs-exposed workers, and decreased with the increase of RCFs exposure. The result corresponded with previous studies and showed that RCFs exposure might cause lung function injury [25,26,27]. 

Transforming growth factor-β1 (TGF-β1) is a multifunctional protein that regulates cell proliferation, tissue repair, angiogenesis, and tumor development. It stimulates pulmonary fibrosis and inflammation, and plays an important role in the process of interstitial lung disease (ILD) [28,29,30,31]. In our study, workers that were exposed to high fiber level and high total dust level had significant higher TGF-β1 level than other workers. Although there was no significant correlation between TGF-β1 and lung function indicators, TGF-β1 still have positive relationship with respirable fiber exposure level. The association between TGF-β1 and respirable fiber exposure level may not be affected by gender, but was only significant in ever-smokers, which indicated that smoking might aggravate the adverse effects on respiratory system of RCFs exposure. This phenomenon suggested that RCFs exposure might increase the level of TGF-β1, especially in smoking workers. In addition, it was necessary to analyze the correlation between lung function indicators and TGF-β1 in bronchoalveolar lavage fluid (BALF) if possible. 

Serum ceruloplasmin (CP) is a glycoprotein containing copper, reflecting the level of oxidative stress and playing a critical role in the process of collagen fibrils formation, which is associated with the fiber lesions in pneumoconiosis [32,33]. Our study showed that CP increased in RCFs-exposed workers, especially in workers that were exposed to high fiber level and high total dust level. The level of CP was negatively correlated with lung function indicators (FVC and FEV_1_), and was positively related to respirable fiber exposure level. So, CP might be applied as biomarkers of lung inflammatory and fibrotic changes that are caused by RCFs exposure. Besides, gender may also influence the relationship between CP and RCFs exposure. In female workers, there was a positive relation between CP and respirable fiber exposure level, while in male workers there was not, which indicated that female workers might be more sensitive to RCFs exposure than male workers. The level of CP may also be affected by smoking status, especially in male workers. However, even without the assistance of smoking, the level of CP still showed a significant association with respirable fiber exposure level in never-smoking workers. 

In this study, we adopted two methods to describe the exposure features of RCFs-related workers (total dust concentration (mg/m^3^) and repirable fiber concentration (f/cm^3^)) to figure out which RCFs measuring method has a closer association with biomarkers. From Table 3, we can infer that respirable fiber exposure level might have closer association with the biomarkers than total dust exposure level. Further studies should be conducted to analyze the association between the physicochemical characteristic of RCFs and the health effects. Due to technical restrictions we could only measure the concentration of RCFs in the air of the workplace in this study. Nevertheless, since air samples were collected at the respiratory zone of workers and gauze masks could not filter RCFs, concentration of RCFs in the air could approximately reflect the actual exposure level of workers in this study. Furthermore, according to the findings, better personal protective equipment, including RCFs-filter mask, should be employed in this factory, especially in the module workplace. 

Generally, previous epidemiologic studies assessed respiratory symptoms, lung function test, and chest X-ray test to reflect the health effects of RCFs exposure. This is the first study that investigated respiratory injury related biomarkers in RCFs-exposed workers and explored the association between lung function indicators and biomarkers in Chinese RCFs workers. It lays a basis for using biomarkers to monitor early respiratory injures in RCFs-exposed workers.

The limitation of this study is that it failed to associate biomarkers with exposure years and other physical effects, like respiratory symptoms and chest X-ray changes. In order to estimate the sensitivity and specificity of these biomarkers, further research should be conducted to determine the correlation between changes in the levels of these biomarkers and actual fibrosis in a RCFs-exposed cohort, so as to demonstrate the utility of these potential indicators of pulmonary inflammation and fibrosis. The other limitation of this study is that the exposure to RCFs for subjects without RCFs exposure were not measured. Since the subjects of the control group were almost free from RCFs exposure and were analyzed as the lowest exposure level group, this will not have obvious influence on our results and conclusions. However, in order to make the research more persuasive, we will measure the exposure to RCFs of controls groups in further researches.

## 5. Conclusions

Occupational RCFs exposure can impair lung ventilation function and may have the potential to cause pulmonary inflammation and fibrosis. TGF-β1 and CP might be used as sensitive and noninvasive biomarkers to detect lung injury in occupational RCFs-exposed workers. Respirable fiber concentration can better reflect occupational RCFs exposure and related respiratory injuries. 

## Figures and Tables

**Table 1:** Fiber and total dust concentration in different refractory ceramic fibers (RCFs)-related workshops.

RCFs-Related Workshops	Number of Air Samples (Pair)	C*_TWA_*_, 40 h_ of Fiber (f/cm^3^)		C*_TWA_*_, 40 h_ of Total Dust (mg/m^3^)		Number of Workers	Exposure Feature
		Arithmetic Mean	Range	Arithmetic Mean	Range		
Manufacturing workshop 1	15	0.12	0.08 to 0.27	3.14	1.70 to 4.95	24	Low fiber, low total dust
Manufacturing workshop 2	25	0.13	0.09 to 0.19	1.72	1.05 to 2.94	27	Low fiber, low total dust
Manufacturing workshop 3	15	0.24	0.02 to 0.49	2.64	0.63 to 4.64	25	Low fiber, low total dust
Manufacturing workshop 4	15	0.20	0.11 to 0.30	2.68	1.70 to 3.59	25	Low fiber, low total dust
Processing workshop	26	0.20	0.10 to 0.40	7.06	5.53 to 16.12	39	Low fiber, high total dust
Module workshop	24	0.79	0.72 to 1.04	8.20	5.47 to 13.82	32	High fiber, high total dust

**Table 2:** General information of different RCFs-exposed subgroups and control group.

Parameter	Index	Control Group (*n* = 171)	Low Fiber, Low Dust (LL) (*n* = 101)	Low Fiber, High Dust (LH) (*n* = 39)	High Fiber, High Dust (HH) (*n* = 32)	*F*	*χ*^2^	*p* Value
Age (year)	Mean ± SD	33.20 ± 6.41	31.88 ± 5.84	31.77 ± 5.15	37.88 ± 5.49 *^,#^	8.711		<0.001
Gender	*n* (%)						19.923	<0.001
Male		125 (73.1)	62 (61.4)	14 (35.9)	20 (62.5)			
Female		46 (26.9)	39 (38.6)	25 (64.1)	12 (37.5)			
Smoking habits	*n* (%)							
Never-smokers		102 (59.6)	66 (65.3)	32 (82.1)	21 (65.6)		7.047	0.070
Ever-smokers		69 (40.4)	35 (34.7)	7 (17.9)	11 (34.4)			
TGF-β1 (ng/mL)	Mean ± SD	0.99 ± 0.29	1.06 ± 0.34	1.10 ± 0.43	1.21 ± 0.49 *	4.055		0.008
CP (U/L)	Mean ± SD	97.90 ± 35.01	106.17 ± 37.43	106.99 ± 33.16	115.25 ± 32.44 *	2.770		0.042
FVC (percentage of predicted value)	Mean ± SD	85.42 ± 13.36	80.58 ± 10.28 *	79.66 ± 13.11 *	78.95 ± 8.92 *	8.960		0.002
FEV_1_ (percentage of predicted value)	Mean ± SD	86.42 ± 13.04	83.09 ± 9.56 *	81.61 ± 11.55 *	81.54 ± 8.36 *	3.031		0.030
FEV_1_/FVC	Mean ± SD	87.60 ± 7.33	86.08 ± 4.80	85.77 ± 6.24	84.73 ± 6.35 *	2.308		0.077

* *p* < 0.05 when compared with the control group with LSD-*t* test, ^#^ *p* < 0.05 when compared with other RCFs-exposed subgroup with LSD-*t* test. LSD-*t* test, least significant difference *t* test.

**Table 3:** Influence factors of biomarkers analyzed by multiple linear regressions.

Biomarkers	Groups	Variables in the Equation	B	*p* Value
**TGF-β1**	All subjects	Respirable fiber exposure level	0.103	0.001
	Ever-smokers	Respirable fiber exposure level	0.245	<0.001
	Never-smokers	-		
	Male	Respirable fiber exposure level	0.098	0.010
	Female	Respirable fiber exposure level	0.106	0.043
**CP**	All subjects	Respirable fiber exposure level	8.027	0.007
		Gender	−14.710	0.001
		Smoking habit	13.926	0.002
	Ever-smokers	-		
	Never-smokers	Respirable fiber exposure level	7.238	0.048
		Gender	−15.751	0.001
	Male	Smoking habit	14.334	0.004
	Female	Respirable fiber exposure level	10.006	0.048

## Supplementary Materials

Supplementary File 1

## Appendix A

**Table A1:** Equation of calculating individual predicted value of lung function indicators developed by European Respiratory Society (ERS) in 1993.

Indicators	Equations	
	Male	Female
FVC	0.0576 × (Height × 100) − 0.026 × Age − 4.34	0.0443 × (Height × 100) − 0.026 × Age − 2.89
FEV_1_	0.0395 × (Height × 100) − 0.025 × Age − 2.60	0.0430 × (Height × 100) − 0.029 × Age − 2.49

**Table A2:** Variable assignment of independent variables of multiple linear regression analysis.

Variables	Assignment
Fiber exposure level	0 = Control group, 1 = Low exposure level, 2 = High exposure level
Total dust exposure level	0 = Control group, 1 = Low exposure level, 2 = High exposure level
Smoking status	0 = Never-smokers, 1 = Ever-smokers
Gender	0 = Female, 1 = Male
Age, years	Continuous variable
